# Arterial Stiffness Is More Associated with Albuminuria than Decreased Glomerular Filtration Rate in Patients with Type 2 Diabetes Mellitus: The REBOUND Study

**DOI:** 10.1155/2017/7047909

**Published:** 2017-08-16

**Authors:** Jong Ho Kim, Sang Soo Kim, In Joo Kim, Bo Hyun Kim, Ja Young Park, Chang Won Lee, Ji Hye Suk, Sun Hae Shin, Sung Pyo Son, Min Chul Kim, Jun Hyeob Ahn, Kwang Jae Lee, Min Jung Kwon, Soon Hee Lee, Jeong Hyun Park

**Affiliations:** ^1^Department of Internal Medicine, Pusan National University Hospital, Busan, Republic of Korea; ^2^Department of Internal Medicine, Busan St. Mary's Medical Center, Busan, Republic of Korea; ^3^Department of Internal Medicine, Woori Medical Clinic, Busan, Republic of Korea; ^4^Department of Internal Medicine, Bongseng Memorial Hospital, Busan, Republic of Korea; ^5^Department of Internal Medicine, Ilsin Christian Hospital, Busan, Republic of Korea; ^6^Department of Internal Medicine, Good Moonhwa Hospital, Busan, Republic of Korea; ^7^Department of Internal Medicine, Daedong Hospital, Busan, Republic of Korea; ^8^Department of Internal Medicine, College of Medicine, Pusan Paik Hospital, Inje University, Busan, Republic of Korea

## Abstract

**Aim:**

The aim of this study was to evaluate the association between arterial stiffness and albuminuria and glomerular filtration rate (GFR) in patients with type 2 diabetes mellitus.

**Methods:**

This multicenter cohort study analyzed 2613 patients with type 2 diabetes. Brachial-ankle pulse wave velocity (baPWV) was used as a noninvasive marker of arterial stiffness. Additionally, the patients were categorized into four groups according to their albumin-to-creatinine ratio (ACR, normoalbuminuria versus albuminuria) and estimated GFR (eGFR, <60 mL/min/1.73 m^2^ versus ≥60 mL/min/1.73 m^2^).

**Results:**

A univariate analysis revealed that maximal baPWV was significantly associated with both the ACR (*r* = 0.297, *P* < 0.001) and eGFR (*r* = −0.220, *P* < 0.001). A multivariate analysis adjusted for significant clinical variables and eGFR showed that baPWV remained significantly correlated with the ACR (*r* = 0.150, *P* < 0.001). Also, baPWV was correlated positively with the ACR in patients with an eGFR ≥ 60 mL/min/1.73 m^2^ (*r* = 0.146, *P* < 0.001). However, baPWV was not correlated with eGFR after adjustment for significant clinical variables.

**Conclusions:**

The present findings indicate that arterial stiffness is more associated with albuminuria than a decrease in GFR in patients with type 2 diabetes mellitus.

## 1. Introduction

Diabetic nephropathy is one of the most serious microvascular complications that influence the mortality of diabetic patients [1]. It is estimated that 20–40% of diabetic patients are affected by this disorder, which manifests clinically as albuminuria or as a reduced glomerular filtration rate (GFR) [2–4]. Varying degrees of increased arterial stiffness are associated with different stages of chronic kidney disease (CKD) [5] and are also an independent risk factor for cardiovascular disease (CVD) and mortality [6]. Most studies have shown that arterial stiffness is independently associated with the two main components of CKD, albuminuria and a reduced GFR [7–10]. Although some studies have found that these associations can be identified in patients with type 2 diabetes [11–14], there are conflicting data regarding these relationships [15].

One previous study reported that up to 25% of patients with either type 1 or type 2 diabetes exhibit reduced renal function under conditions of a normal albumin excretion rate (AER; <20 *μ*g/min) [16], and subsequent studies have supported the dissociation between a decreased GFR and increased albuminuria in patients with type 2 diabetes [17–19]. Thus, the traditional paradigm of diabetic kidney disease has been challenged, and changes in the AER and GFR are being accepted as interdependent rather than essential manifestations of diabetic CKD. The discordance between changes in the AER and GFR has brought about a search for new markers that can accurately identify diabetic patients at risk of a declining GFR independent of gradual increases in AER.

The relationship between CVD and brachial-ankle pulse wave velocity (baPWV) in Korean patients with type 2 diabetes was previously investigated to assess the prognostic efficacy of baPWV for cardiovascular morbidity and mortality [20]. The primary aims of the subanalysis of that study conducted here were to determine whether arterial stiffness is associated with diabetic nephropathy and to evaluate the associations of baPWV with albuminuria and GFR as key factors underlying the development and progression of type 2 diabetic nephropathy. Additionally, whether or not baPWV has different associations with albuminuria and GFR was evaluated.

## 2. Methods

The REBOUND study was designed as a multicenter prospective observational study for the assessment of the association between baPWV and CVD in patients with type 2 diabetes [20]. Briefly, the REBOUND study was conducted from December 2008 to December 2010 at eight general hospitals in Busan, Korea. That study consecutively recruited 3058 patients with type 2 diabetes 30 years of age and older and measured their baPWV values, as a noninvasive marker of arterial stiffness, based on the procedures of the outpatient endocrinology departments of each hospital. The exclusion criteria were as follows: a low ankle-brachial index (ABI; <0.9), severe symptoms and/or signs of CVD, a history of acute myocardial infarction, stroke, or hospitalization for heart failure within 3 months, and chronic renal disease (serum creatinine levels > 2.0 mg/dL).

### 2.1. Data Collection

All data were collected from the medical records and physical examinations of the patients. Laboratory data obtained within the 3 months prior to enrollment were collected from available sources, and blood samples intended for biochemical analyses were collected after the participants fasted for at least 8 hours. Using an automatic waveform analyzer (VP-2000, Colin, Komaki, Japan), baPWV was measured automatically by the brachial-ankle distance (*L* = 0.5934 × height [cm] + 14.4014) divided by the pulse wave time interval between the brachial region and ankle (Δ*T*) while the participants were kept supine for 5 minutes [21]. The right and left baPWV values were obtained, and the largest value was determined as the maximum baPWV (M-baPWV). The estimated GFR (eGFR) levels were determined using the modification of diet in renal disease (MDRD) equation: MDRD = 186 × (serum creatinine [mg/dL])^–1.154^ × (age in years)^–0.203^ [22]; an adjustment factor of 0.742 was used for women. Albuminuria (microalbuminuria and overt albuminuria) was defined based on the albumin-to-creatinine ratio (ACR; ≥30 mg/g creatinine) using random spot urine testing. The prevalence of retinopathy, neuropathy, and CVD were investigated based on medical history. Diabetic retinopathy was detected during an eye examination that includes fundus photography or ophthalmoscopy. Diabetic neuropathy was diagnosed based on symptoms, medical history, and a physical examination. CVD includes coronary heart disease, cerebrovascular disease, peripheral arterial disease, rheumatic heart disease, congenital heart disease, deep vein thrombosis, and pulmonary embolism.

### 2.2. Population and Statistical Analysis

Of the 3058 patients enrolled in the REBOUND study, 445 were excluded from the analysis due to a violation of the inclusion and/or exclusion criteria or because there were no available data for baPWV, eGFR, or ACR. In the present subanalysis, the data of 2613 patients were analyzed. The patients were categorized into four groups according to the ACR (normoalbuminuria versus albuminuria) and eGFR (<60 mL/min/1.73 m^2^ versus ≥60 mL/min/1.73 m^2^).

The statistical package SPSS version 15.0 (SPSS Inc., Chicago, IL, USA) was used for all data analyses. The data are presented as means ± standard deviations (SD) for normally distributed variables and as medians (interquartile ranges) for nonparametric variables. The distribution of the continuous variables was examined for skewness and kurtosis, and the logarithm-transformed values were used for the analysis. Differences among groups were analyzed by analysis of variance (ANOVA) followed by a Bonferroni's test for parametric values and the Kruskal-Wallis test for nonparametric values. Pearson's chi-squared (*χ*^2^) test was applied to analyze categorical variables. Pearson's correlation coefficient test was used to assess the relationship between two variables. Multivariate regression analyses using either ACR or eGFR as the dependent variable and baPWV as the independent variable were conducted, and several models were used to adjust for confounding variables. A two-tailed *P* value < 0.05 was considered to indicate statistical significance for all statistical tests.

### 2.3. Ethics Statement

The protocol for the present study was approved by the institutional review boards of each hospital, including that of Pusan National University Hospital (numbers 2009041 and 20132131), and informed consent was obtained from all patients for which identifying information is included in this article.

## 3. Results

### 3.1. Patient Demographics

The demographic characteristics of the patients are shown in Table 1. The mean age of the entire population was 59.6 ± 10.7 years (range: 30–89 years), 43.4% of the population was male, 56.6% of the population was female, and the mean duration of diabetes was 9.1 ± 6.9 years. The average body mass index (BMI) was 24.9 ± 3.4 kg/m^2^, the average waist circumference was 88.8 ± 8.7 cm, the average glycated hemoglobin (HbA1c) level was 7.6 ± 1.6% (59.8 ± 18.0 mmol/mol), and the prevalence rates of albuminuria and CKD of stage 3 or greater were 31.2% and 18.3%, respectively. The patients were categorized into four groups according to albuminuria status and eGFR: those with an eGFR ≥ 60 mL/min/1.73 m^2^ and normoalbuminuria (*n* = 1575), those with an eGFR < 60 mL/min/1.73 m^2^ and normoalbuminuria (*n* = 223), those with an eGFR ≥ 60 mL/min/1.73 m^2^ and albuminuria (ACR ≥ 30 mg/g creatinine; *n* = 559), and those with an eGFR < 60 mL/min/1.73 m^2^ and albuminuria (*n* = 256).

Among the normoalbuminuric patients, all variables except for diastolic blood pressure (DBP), heart rate, low-density lipoprotein (LDL) cholesterol level, and the ABI significantly differed between the two groups according to the eGFR (Table 1). The M-baPWV was higher in patients with an eGFR < 60 mL/min/1.73 m^2^ than in patients with an eGFR ≥ 60 mL/min/1.73 m^2^. Among the albuminuric patients, there were no differences in BMI, waist circumference, DBP, heart rate, HbA1c, LDL cholesterol, triglyceride, and ABI levels according to eGFR between the two groups. However, the M-baPWV was higher in patients with an eGFR < 60 mL/min/1.73 m^2^ than in patients with an eGFR ≥ 60 mL/min/1.73 m^2^. Among both normoalbuminuric and albuminuric patients, CVD was more prevalent in patients with an eGFR < 60 mL/min/1.73 m^2^ than in patients with an eGFR ≥ 60 mL/min/1.73 m^2^ (Table 2). Additionally, two major complications, neuropathy and retinopathy, were more frequently observed in patients with an eGFR < 60 mL/min/1.73 m^2^ than in patients with an eGFR ≥ 60 mL/min/1.73 m^2^.

### 3.2. Associations of baPWV with the ACR and eGFR

A univariate regression analysis of the entire population revealed that baPWV was significantly associated with the ACR (model 1; Table 3) and eGFR (model 1; Table 4). The patients were divided into four groups according to quartiles of M-baPWV levels, and comparison of clinical characteristics was shown in Supplementary Table 1 available online at https://doi.org/10.1155/2017/7047909. After adjusting for age, sex, and significant clinical variables such as BMI, duration of diabetes, SBP, pulse pressure, heart rate, smoking, alcohol consumption, HbA1c, HDL cholesterol, hsCRP, insulin treatment, and RAS inhibitors, baPWV was significantly correlated with the ACR (models 2 and 3; Table 3). After additional adjustments for eGFR, baPWV remained significantly associated with the ACR (*r* = 0.150, *P* < 0.001). When the patients were stratified by eGFR, baPWV was positively correlated with the ACR in the final model after adjusting for several clinical variables and eGFR in patients with an eGFR ≥ 60 mL/min/1.73 m^2^ (*r* = 0.146, *P* < 0.001) and <60 mL/min/1.73 m^2^ (*r* = 0.091, *P* = 0.071).

However, the significant association of baPWV with eGFR among all patients was lost after adjusting for clinical variables (models 3 to 4; Table 4). When the patients were stratified by albuminuria status, baPWV was significantly associated with eGFR in the univariate analysis (model 1; Table 4), but this significant relationship with eGFR was lost after adjusting for clinical variables in both the normoalbuminuria and albuminuria groups (models 2–4; Table 4). These results were the same when the albuminuria group was divided into the microalbuminuria group and the macroalbuminuria group (Supplementary Table 2).

## 4. Discussion

Although several cross-sectional studies have demonstrated that CKD is correlated with aortic stiffening [5, 7], the association of aortic stiffness with CKD in patients with diabetes has received less attention. In the present study, compared with a decline in GFR, arterial stiffness was more associated with albuminuria in patients with type 2 diabetes mellitus. However, arterial stiffness was not associated with GFR in both the normoalbuminuric and albuminuric patients with type 2 diabetes after adjusting for several significant clinical variables.

Several previous studies are in agreement with the present data regarding the relationship between arterial stiffness and the ACR and/or GFR in diabetic patients. A study of Chinese middle-aged adults demonstrated that albuminuria is strongly related to arterial stiffness (measured using baPWV) and that this relationship is enhanced in subjects with hypertension, diabetes, or macroalbuminuria [12]. Similarly, a Japanese longitudinal study found that aortic stiffness (measured using carotid-femoral pulse wave velocity (cfPWV)) is related to incidental albuminuria and the rate of GFR decline in patients with type 2 diabetes [13]. On the other hand, pulse pressure (PP) is often used to measure arterial stiffness in the clinical field. In the Veterans Affairs Diabetes Trial (VADT) subanalysis, Anderson et al. [14] reported accelerated ACR deterioration in subjects with a relatively high PP and attenuated ACR deterioration in subjects with a relatively low PP. These authors also found that arterial stiffness (defined by a PP ≥ 60 mmHg) was significantly associated with a worsening ACR, but not with a worsening eGFR [14]. There is also controversy regarding the relationships between PP and ACR and/or GFR. In the Australian Diabetes, Obesity, and Lifestyle (AusDiab) study, a higher PP (defined as ≥61 mmHg) was a significant risk factor for a decline in eGFR, but not albuminuria, over a 5-year period, especially in individuals with type 2 diabetes [15].

The mechanisms underlying the relationship between a greater degree of arterial stiffness and heightened albuminuria or a decreased GFR in diabetic patients have not been established. As arterial stiffness increases, the myocardium and kidneys are exposed to higher systolic pressures and greater pressure fluctuations resulting in myocardial hypertrophy and fibrosis, renal microvascular damage, and an increased risk of renal dysfunction [23]. The combination of endothelial dysfunction and inflammation may be plausible mechanisms linking aortic stiffness and CKD [13]. Additionally, the afferent arteriole branch from the renal artery is a short vessel exposed to high pressures and, therefore, must maintain a strong arteriolar tone to provide a high pressure gradient over a short distance [13]. It is possible that these vessels are controlled by the hemodynamics of large arteries rather than small vessels in the peripheral circulation. As a result, the stiffness of large arteries may directly increase PP, especially in these vessels, and lead to glomerular or tubular damage via elevated intrarenal PP [23]. In turn, this may cause renal microvascular dysfunction, including albuminuria or a reduction in GFR.

One strength of the present study was the use of a large population-based cohort that included 2613 patients from eight general hospitals. Furthermore, arterial stiffness was evaluated by baPWV, which may be more applicable in general practice because its measurement is automated and easier to perform compared to that of cfPWV [24]. However, there were also several limitations to the present study. First, the present findings were based on a cross-sectional design rather than longitudinal observations. Thus, further investigations are required to find out whether patients with elevated excreted urinary markers (kidney injury molecule-1 (KIM-1), neutrophil gelatinase-associated lipocalin (NGAL), and liver-type fatty acid-binding protein (L-FABP)) and normoalbuminuria are more vulnerable to a decline in GFR or the progression of albuminuria. Our research group is now working to clear up this issue. Second, a homogeneous population was used in the present study because it was hospital based. Besides, the REBOUND study was designed to show the relationship between CVD and baPWV in patients with type 2 diabetes. Therefore, the subjects with low ABI, severe CVD, and elevated serum creatinine greater than 2.0 mg/dL, which adversely affect survival [25], were excluded. As a result, the generalization of the present findings to all patients with type 2 diabetes mellitus may be limited. Third, in order to determine urine ACRs, random spot urine samples were collected at only one time point, although urine samples were obtained from patients without illness or prior kidney diseases other than diabetic nephropathy. Fourth, it is likely that the reduction in muscle mass in elderly patients with type 2 diabetes may have influenced eGFR levels and led to a misclassification of changes in eGFR levels as well as underestimation of the relationship between aortic stiffness and decreased GFR. Fifth, the results of the present study were not novel, but it is worthwhile to show that arterial stiffness has different associations with albuminuria and GFR in a large population-based cohort.

In conclusion, the present findings demonstrated that, compared with a decrease in the GFR, arterial stiffness was more associated with albuminuria in patients with type 2 diabetes mellitus. The effect of arterial stiffening on albuminuria or a decreased GFR needs to be analyzed in future large longitudinal studies.

## Supplementary Material

Supplementary Table 1. Comparison of clinical characteristics according to M-baPWV quartiles. Supplementary Table 2. Multivariate regression analyses with eGFR as a dependent variable and baPWV as an independent variable.

## Figures and Tables

**Table 1:** Comparison of clinical characteristics according to albuminuria and eGFR group.

Characteristics	Normoalbuminuria		*P* value	Albuminuria		*P* value
	eGFR ≥ 60 (*n* = 1575)	eGFR < 60 (*n* = 223)		eGFR ≥ 60 (*n* = 559)	eGFR < 60 (*n* = 256)	
Age, years	58.0 ± 10.1	67.2 ± 9.2	<0.001	58.6 ± 11.3	65.1 ± 10.0	<0.001
Sex, male/female	685/890	58/165	<0.001	286/273	104/152	0.005
BMI, kg/m^2^	24.7 ± 3.2	25.2 ± 3.0	0.016	25.4 ± 3.9	25.0 ± 3.4	0.318
Waist circumference, cm	88 ± 8	91 ± 9	<0.001	91 ± 10	90 ± 9	0.729
Duration of diabetes, years	7.9 ± 7.0	10.1 ± 6.8	<0.001	10.0 ± 7.7	13.3 ± 7.8	<0.001
SBP, mmHg	126 ± 14	131 ± 18	0.005	132 ± 17	140 ± 22	<0.001
DBP, mmHg	78 ± 9	76 ± 10	0.555	80 ± 10	78 ± 12	0.614
Pulse pressure, mmHg	48 ± 11	55 ± 14	<0.001	52 ± 13	61 ± 16	<0.001
Heart rate, bpm	74 ± 11	75 ± 12	0.568	78 ± 12	76 ± 12	0.093
HbA1c, %	7.4 ± 1.5	7.5 ± 1.5	0.012	8.1 ± 1.8	7.8 ± 1.8	0.771
HbA1c, mmol/mol	57.6 ± 16.8	58.4 ± 16.8	0.012	65.4 ± 19.5	62.2 ± 19.7	0.771
eGFR, mL/min/1.73 m^2^	87.3 ± 21.5	52.1 ± 8.2	<0.001	85.6 ± 21.9	45.1 ± 12	<0.001
ACR, mg/g^∗^	6.2 (3.3–12.1)	7.4 (4.3–14.2)	0.033	82.1 (44.8–193.9)	220.9 (74.7–827.6)	<0.001
LDL cholesterol, mg/dL	93 ± 33	91 ± 32	0.564	95 ± 31	92.9 ± 36	0.824
HDL cholesterol, mg/dL	49 ± 12	47 ± 12	0.001	48 ± 13	44 ± 12	<0.001
Triglyceride, mg/dL^∗^	115 (82–164)	116 (89–166)	0.007	130 (93–200)	133 (98–182)	0.140
hsCRP, mg/dL^∗^	0.11 (0.05–0.45)	0.19 (0.06–1.00)	<0.001	0.19 (0.08–0.91)	0.32 (0.09–1.23)	0.017
M-baPWV, cm/sec^∗^	1531 (1363–1725)	1708 (1490–2006)	0.002	1677 (1443–1934)	1878 (1615–2161)	0.005
Right ABI	1.11 ± 0.09	1.10 ± 0.10	0.094	1.09 ± 0.12	1.08 ± 0.13	0.574
Left ABI	1.11 ± 0.09	1.11 ± 0.11	0.786	1.10 ± 0.10	1.09 ± 0.13	0.587
Smoking, %	21.6	8.7	<0.001	26.8	15.9	0.001
Alcohol consumption, %	28.5	16.8	<0.001	33.3	19.1	<0.001
Insulin treatment, %	19.5	28.3	0.002	35.1	52.3	<0.001
RAS inhibitors, %	41.6	52.5	0.001	62.1	71.1	0.012
Lipid lowering agent, %	58.5	65.9	0.036	64.2	61.3	0.426
Antiplatelet agent, %	60.6	66.4	0.096	61.5	64.1	0.490

Values are presented as mean ± SD for parametric variables and median (interquartile range) for nonparametric variables. *t*-test for age; chi-square test for categorical variables; age and sex-adjusted ANCOVA for all other continuous variables; ^∗^logarithm-transformed values were used for comparison. M-baPWV: maximum brachial-ankle pulse wave velocity; BMI: body mass index; SBP: systolic blood pressure; DBP: diastolic blood pressure; ABI: ankle-brachial index; LDL: low-density lipoprotein; HDL: high-density lipoprotein; HbA1c: hemoglobin A1c; hsCRP: high-sensitivity C-reactive protein; eGFR: estimated glomerular filtration rate.

**Table 2:** The prevalence of chronic complications according to the albuminuria and eGFR group.

Characteristics	Normoalbuminuria		*P* value	Albuminuria		*P* value
	eGFR ≥ 60 (*n* = 1575)	eGFR < 60 (*n* = 223)		eGFR ≥ 60 (*n* = 559)	eGFR < 60 (*n* = 256)	
Cardiovascular disease	7.7	18.6	<0.001	6.5	14.9	<0.001
Coronary artery disease	5.8	16.2	<0.001	4.4	11.8	<0.001
Cerebrovascular disease	2.0	4.4	0.029	2.2	2.6	0.711
Peripheral artery disease	0.8	1.5	0.315	0.4	1.3	0.201
Neuropathy	38.7	56.3	<0.001	52.2	65.9	<0.001
Retinopathy	13.4	21.4	0.003	28.9	48.6	<0.001

eGFR: estimated glomerular filtration rate.

**Table 3:** Multivariate regression analyses with ACR as a dependent variable and baPWV as an independent variable.

Model	All (*n* = 2613)		eGFR ≥ 60 (*n* = 2134)		eGFR < 60 (*n* = 479)	
	Standard *β*	*P* value	Standard *β*	*P* value	Standard *β*	*P* value
1	0.297	<0.001	0.251	<0.001	0.216	<0.001
2	0.347	<0.001	0.298	<0.001	0.364	<0.001
3	0.152	<0.001	0.145	<0.001	0.131	0.013
4	0.150	<0.001	0.146	<0.001	0.091	0.071

Model 1: crude; model 2: adjusted for age and sex; model 3: adjusted for significant clinical parameters including BMI, duration of diabetes, SBP, pulse pressure, heart rate, smoking, alcohol consumption, HbA1c, HDL cholesterol, hsCRP, insulin treatment, and RAS inhibitors; model 4: adjusted for eGFR; ACR: albumin-to-creatinine ratio; baPWV: brachial-ankle pulse wave velocity; eGFR: estimated glomerular filtration rate.

**Table 4:** Multivariate regression analyses with eGFR as a dependent variable and baPWV as an independent variable.

Model	All (*n* = 2613)		Normoalbuminuria (*n* = 1798)		Albuminuria (*n* = 815)	
	Standard *β*	*P* value	Standard *β*	*P* value	Standard *β*	*P* value
1	−0.220	<0.001	−0.141	<0.001	−0.245	<0.001
2	−0.071	0.001	0.025	0.357	−0.090	0.017
3	−0.013	0.620	0.011	0.736	0.013	0.769
4	−0.013	0.635	0.012	0.711	0.050	0.243

Model 1: crude; model 2: adjusted for age and sex; model 3: adjusted for significant clinical parameters including BMI, duration of diabetes, SBP, pulse pressure, heart rate, smoking, alcohol consumption, HbA1c, HDL cholesterol, hsCRP, insulin treatment, and RAS inhibitors; model 4: adjusted for ACR; ACR: albumin-to-creatinine ratio; baPWV: brachial-ankle pulse wave velocity; eGFR: estimated glomerular filtration rate.
