# Supplementary material for: Arterial Stiffness Is More Associated with Albuminuria than Decreased Glomerular Filtration Rate in Patients with Type 2 Diabetes Mellitus: The REBOUND Study
Source: J Diabetes Res. 2017 Aug 16;2017:7047909. doi: 10.1155/2017/7047909 (PMC5603750; doi:10.1155/2017/7047909)
Supplement: Supplementary file 1 — Supplementary Table 1. Comparison of clinical characteristics according to M-baPWV quartiles. Supplementary Table 2. Multivariate regression analyses with eGFR as a dependent variable and baPWV as an independent variable. [file 7047909.f1.docx]

**Supplementary Table 1.** Comparison of clinical characteristics according to M-baPWV quartiles

|  | Q1 (n = 655) | Q2 (n = 653) | Q3 (n = 653) | Q4 (n = 652) | P value |
| --- | --- | --- | --- | --- | --- |
| Age, years | 52.0 ± 9.8 | 57.4 ± 9.2 | 61.7 ± 8.8 | 67.3 ± 8.6 | <0.001 |
| Sex, male/female | 323/332 | 302/351 | 269/384 | 239/413 | <0.001 |
| BMI, kg/m^2^ | 25.2 ± 3.7 | 25.0 ± 3.3 | 25.0 ± 3.1 | 24.6 ± 3.3 | 0.045 |
| Waist circumference, cm | 88 ± 9 | 89 ± 8 | 89 ± 8 | 89 ± 9 | 0.163 |
| Duration of diabetes, years | 6.6 ± 5.1 | 8.0 ± 6.0 | 9.3 ± 6.9 | 12.2 ± 8.1 | <0.001 |
| SBP, mmHg | 120 ± 13 | 127 ± 14 | 130 ± 15 | 139 ± 19 | <0.001 |
| DBP, mmHg | 76 ± 9 | 78 ± 9 | 79 ± 10 | 80 ± 11 | <0.001 |
| Pulse pressure, mmHg | 45 ± 9 | 49 ± 11 | 52 ± 12 | 59 ± 14 | <0.001 |
| Heart rate, bpm | 73 ± 11 | 74 ± 11 | 77 ± 12 | 78 ± 12 | <0.001 |
| HbA1c, % | 7.7 ± 1.9 | 7.5 ± 1.6 | 7.6 ± 1.5 | 7.7 ± 1.6 | 0.104 |
| eGFR, mL/min/1.73m^2^ | 81.6 (70.3-94.6) | 79.5 (67.4-92.8) | 77.8 (66.0-92.3) | 68.6 (57.0-83.7) | <0.001 |
| ACR, mg/g* | 7.2 (3.6-22.5) | 9.4 (4.1-28.5) | 12.5 (5.1-45.3) | 28.3 (7.9-124.8) | <0.001 |
| LDL cholesterol, mg/dL | 94 ± 34 | 93 ± 31 | 93 ± 33 | 94 ± 33 | 0.903 |
| HDL cholesterol, mg/dL | 48 ± 12 | 49 ± 13 | 48 ± 12 | 48 ± 13 | 0.395 |
| Triglyceride, mg/dL* | 117 (82-169) | 117 (83-172) | 124 (88-176) | 123 (90-166) | 0.123 |
| hsCRP, mg/dL* | 0.12 (0.05-0.51) | 0.13 (0.05-0.46) | 0.15 (0.06-0.82) | 0.19 (0.07-0.80) | 0.002 |
| M-baPWV, cm/sec* | 1292 (1228-1350) | 1499 (1451-1548) | 1704 (1650-1766) | 2046 (1931-2259) | <0.001 |
| Right ABI | 1.10 ± 0.10 | 1.11 ± 0.10 | 1.10 ± 0.10 | 1.09 ± 0.11 | 0.177 |
| Left ABI | 1.10 ± 0.09 | 1.11 ± 0.09 | 1.11 ± 0.12 | 1.10 ± 0.11 | 0.047 |
| Smoking, % | 25.6 | 23.7 | 20.2 | 14.8 | <0.001 |
| Alcohol consumption, % | 33.5 | 31.6 | 25.4 | 19.7 | <0.001 |
| Insulin treatment, % | 23.7 | 20.8 | 24.8 | 37.9 | <0.001 |
| RAS inhibitors, % | 36.0 | 45.9 | 55.1 | 62.1 | <0.001 |

Values are presented as mean ± SD for parametric variables and median (interquartile range) for nonparametric variables.

Quartiles of M-baPWV were 917-1399, 1400–1596, 1597–1840, and 1841-5881.

*logarithm transformed values were used for comparison.

M-baPWV, maximum brachial-ankle pulse wave velocity; BMI, body mass index; SBP, systolic blood pressure; DBP, diastolic blood pres­sure; ABI, ankle brachial index; LDL, low density lipoprotein; HDL, high density lipoprotein; HbA1c, hemoglobin A1c; hs-CRP, high sensitive C-reactive protein; eGFR, estimated glomerula filtration rate.

**Supplementary Table 2.** Multivariate regression analyses with eGFR as a dependent variable and baPWV as an independent variable

| Model | All  (n = 2,613) | |  | Normoalbuminuria  (n = 1,798) | |  | Microalbuminuria  (n = 596) | |  | Macroalbuminuria  (n = 219) | |
| --- | --- | --- | --- | --- | --- | --- | --- | --- | --- | --- | --- |
|  | Standard β | *P* value |  | Standard β | *P* value |  | Standard β | *P* value |  | Standard β | *P* value |
| 1 | -0.220 | <0.001 |  | -0.141 | <0.001 |  | -0.203 | <0.001 |  | -0.205 | 0.002 |
| 2 | -0.071 | 0.001 |  | 0.025 | 0.357 |  | 0.047 | 0.290 |  | -0.081 | 0.270 |
| 3 | -0.013 | 0.620 |  | 0.011 | 0.736 |  | 0.056 | 0.296 |  | 0.049 | 0.550 |
| 4 | -0.013 | 0.635 |  | 0.012 | 0.711 |  | 0.067 | 0.211 |  | 0.046 | 0.566 |

Model 1, crude; Model 2, adjusted for age and sex, Model 3, adjusted for significant clinical parameters including BMI, Duration of diabetes, SBP, Pulse pressure, Heart rate, Smoking, Alcohol consumption, HbA1c, HDL cholesterol, hsCRP, Insulin treatment, and RAS inhibitors; model 4, adjusted for ACR.

ACR, albumin-to-creatinine ratio; baPWV, brachial-ankle pulse wave velocity; eGFR, estimated glomerula filtration rate.
